# In Silico Optimization of a Non-Invasive Optical Sensor for Hemoconcentration Monitoring in Dengue Fever Management

**DOI:** 10.3390/bios16020121

**Published:** 2026-02-13

**Authors:** Murad Althobaiti, Gameel Saleh

**Affiliations:** Biomedical Engineering Department, College of Engineering, Imam Abdulrahman Bin Faisal University, Dammam 31441, Saudi Arabia; mmalthobaiti@iau.edu.sa

**Keywords:** dengue fever, hemoconcentration, non-invasive monitoring, biomedical optics, Monte Carlo simulation, diffuse reflectance spectroscopy

## Abstract

Severe Dengue fever can cause Dengue Hemorrhagic Fever (DHF), a life-threatening condition characterized by plasma leakage and hemoconcentration. A hematocrit (Hct) rise of ≥20% is a key indicator for medical intervention, but current monitoring is invasive and intermittent. This study aims to determine the optimal design parameters for a non-invasive optical sensor to continuously monitor hemoconcentration. We developed a high-fidelity Monte Carlo model of light transport in a multi-layered skin model, with the epidermis set to a 5% melanin volume fraction (Fitzpatrick type II/III). To ensure signal reliability, simulations were conducted with a high photon count ($1\times 10^{8}$ photons), yielding a stochastic (Monte Carlo) signal-to-noise ratio of approximately 36 dB. We simulated diffuse reflectance at four characteristic wavelengths (577 nm, 660 nm, 800 nm—the isosbestic point—, and 940 nm) over source-detector separations of 0.5–8.0 mm. Sensor sensitivity was quantified as the reflectance change for a +25% relative Hct rise (e.g., 42% to 52.5%), mimicking severe hemoconcentration, and its dependence on baseline dermal blood volume fraction (BVF) was investigated. Sensor sensitivity showed a non-linear dependence on BVF, showing a direct correlation with perfusion level, reaching an optimal 6.41% for a robust 5% BVF at 8.0 mm. A dedicated sweep showed that even under low-perfusion shock conditions (1% BVF), the sensor maintains a highly significant sensitivity of 5.71% (also at 8.0 mm), indicating that sensitivity remains high across a physiologically relevant perfusion range. In the analysis, at a robust 5% BVF, the 800 nm wavelength demonstrated superior reliability, with peak sensitivity at 6.41% at 8.0 mm. Visible wavelengths (577 nm and 660 nm) exhibited high theoretical sensitivity, while 940 nm was compromised by water absorption. Based on these findings, a non-invasive optical sensor for hemoconcentration is most effective operating at 800 nm, within the evaluated spectral set, with a source-detector separation of ≥6.0 mm, targeting the deep dermis while minimizing superficial interference. This design provides an optimal balance of tissue penetration, robust sensitivity to Hct changes, and reduced sensitivity to oxygenation-related variability while maintaining signal stability. This work enables the design of a device for continuous monitoring, supporting continuous monitoring of hemoconcentration trends relevant to plasma leakage progression.

## 1. Introduction

Dengue Fever (DF) is a mosquito-borne viral illness that poses a significant global health burden, with millions of cases reported annually. While most infections are self-limiting, a subset of patients may progress to severe forms of the disease, such as Dengue Hemorrhagic Fever (DHF) and Dengue Shock Syndrome (DSS). The defining pathophysiological feature of severe Dengue is an increase in vascular permeability, leading to the leakage of plasma from the intravascular space [1,2]. To diagnose and predict disease severity, clinicians monitor a panel of hematological parameters, including a rising hematocrit, a dropping platelet count (thrombocytopenia), and changes in hemoglobin [3,4].

Of these markers, plasma leakage resulting in hemoconcentration is a primary indicator of progression to DHF. This condition is characterized by an increase in the volume percentage of red blood cells in blood (hematocrit, Hct). According to World Health Organization (WHO) guidelines, a rise in Hct of 20% or more above the patient’s baseline is a major warning sign and a key diagnostic criterion for DHF, necessitating prompt fluid resuscitation to prevent the onset of shock [5]. The degree of hemoconcentration during the plasma leak phase is strongly correlated with disease severity and complications such as ascites and pleural effusion [6]. While thrombocytopenia is also a critical marker [3,4], Hct remains the most direct measure of plasma leakage. Current clinical practice for monitoring Hct relies on periodic, invasive blood draws, which are intermittent, painful for the patient, and present logistical challenges in resource-limited settings.

Non-invasive optical sensing offers a promising alternative for the continuous, real-time monitoring of tissue physiology. Techniques based on near-infrared (NIR) spectroscopy, in particular, have been widely investigated for various medical applications, including the monitoring of blood glucose tissue oxygenation, and hemoglobin concentration [7,8,9]. While continuous non-invasive hemoglobin (Hb) and hematocrit (Hct) monitoring has been explored using optical techniques [9,10], often relying on pulse oximetry or photoplethysmography (PPG) principles, these methods face significant challenges in Dengue management [11,12]. Their reliance on pulsatile signals makes them susceptible to motion artifacts and highly prone to failure during peripheral vasoconstriction in Dengue Shock Syndrome (DSS) [13]. Furthermore, Hct monitoring requires isolating the Hct signal from concurrent changes in blood oxygen saturation (SpO_2_), a problem many commercial devices have yet to solve robustly. Our work, focusing on DC-level Diffuse Reflectance Spectroscopy, aims to bypass these PPG-related limitations and optimize the sensor design for the specific DSS pathology.

Diffuse Reflectance Spectroscopy (DRS) is one such NIR technique that measures light reflected from the skin surface after it has interrogated the underlying tissue layers. The detected signal is modulated by the absorption and scattering properties of the tissue, which are in turn determined by its composition, including the concentration of blood [14]. The challenge in DRS is to isolate the signal from the target layer (e.g., the blood-perfused dermis) from interfering signals originating in superficial layers (e.g., the epidermis). A dual-channel approach, using short and long source-detector separations to differentiate between superficial and deep tissue information, has been proposed as an effective method to address this challenge in applications such as glucose monitoring [15,16] and functional brain imaging [17].

The development of an effective DRS sensor for hemoconcentration requires the careful selection of two key design parameters: the operating wavelength(s) of light and the geometric arrangement of the light source and detector(s) on the skin. The short range of source–detector separation (SDS) of 0.5−8.0 mm used in this study is based on the requirement for a compact, non-invasive wearable form factor (e.g., a forearm or wrist patch). The four wavelengths chosen for this study (577, 660, 800, and 940 nm) were selected to represent the key visible and Near-Infrared optical regimes: the Hb peak, the Hb/HbO_2_ contrast maximum, the Hb/HbO_2_ isosbestic point, and the water absorption peak, respectively. As there is no single universal wavelength for non-invasive hemoglobin measurements, the optimal selection requires a balance between molecular absorption characteristics and tissue penetration depth, and robustness to epidermal melanin absorption (a major limitation for visible-light sensors), necessitating application-specific optimization [18]. Given the high prevalence of dengue in tropical regions with darker skin phototypes, minimizing melanin interference is a critical design requirement. Furthermore, since plasma leakage increases tissue water content (edema), the optimal wavelength must also minimize interference from water absorption. The goal of this study is to determine these optimal parameters for hemoconcentration monitoring through a rigorous computational investigation. We utilize a high-fidelity Monte Carlo simulation, a method established for accurately modeling light transport in complex biological tissues [8,19], to systematically evaluate sensor sensitivity to a clinically significant hemoconcentration event across a range of wavelengths and source-detector separations. This in silico approach allows for the independent manipulation of physiologically coupled variables—such as hematocrit and blood volume fraction—which is often not feasible in clinical studies where these parameters vary simultaneously. This provides a foundational design guide prior to clinical validation.

## 2. Method

A three-dimensional numerical model was developed to simulate light propagation in skin and predict the diffuse reflectance signal. The entire simulation workflow was implemented in MATLAB (R2025a, The MathWorks, Inc., Natick, MA, USA), using the GPU-accelerated Monte Carlo eXtreme (MCX) engine (v2025) [19].

### 2.1. Monte Carlo Simulation Parameters

To ensure statistical reliability and low stochastic noise, a photon count of ${1\times 10}^{8}$ was used for each simulation. A convergence study was performed to determine this parameter by calculating the Signal-to-Noise Ratio (SNR) as a function of photon count (Table 1). The SNR was calculated as [20]:

(1) $${\mathrm{SNR}}_{dB}=20{log}_{10}\left(\frac{\mu }{\sigma }\right)$$

where μ is the mean photon count and σ is the standard deviation across 10 repeated simulation runs. As shown in Table 1, increasing the photon count to $1\times 10^{8}$ yields an SNR of approximately 36 dB (corresponding to a noise level of ~1.6%) at the critical source-detector separation of 6.0 mm. This noise level is significantly lower than the predicted physiological signal changes (>5%), confirming that the simulation parameters are sufficient for reliable detection. It is important to note that this calculated SNR is a statistical measure of the intrinsic Monte Carlo noise and does not represent the electronic or thermal noise of a physical DRS instrument.

The simulation volume was a 200 × 200 × 100 voxel grid, with an isotropic voxel size of 0.1 mm. A time-resolved simulation was run for 10 ns. Given the speed of light in tissue (v≈c/n≈0.22 mm/ps), 10 ns allows photons to traverse pathlengths exceeding 2000 mm, ensuring that the collected signal effectively reaches a steady state without under-sampling late-arriving photons. All simulations were executed on an NVIDIA GeForce GTX 1650 SUPER GPU(Santa Clara, California, CA, USA).

### 2.2. Numerical Tissue Model and Optode Geometry

A multi-layered, voxel-based numerical tissue model was constructed to represent a 20 × 20 × 10 mm^3^ section of human skin. The model consisted of three primary layers with physiologically representative thicknesses: a 0.1 mm epidermis, a 1.5 mm dermis, and an 8.4 mm layer of subcutaneous fat. While dermal thickness varies by anatomical site (e.g., thinner in fingertips), 1.5 mm was chosen as a representative value for potential measurement sites such as the forearm or upper arm [21].

**Figure 1:**
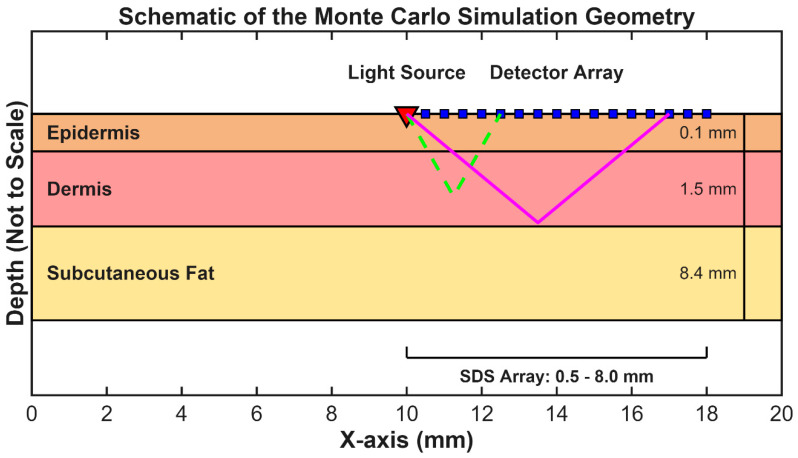
Schematic of the Multi-Layered Skin Phantom and Optode Geometry. The 2D cross-section illustrates the three–layer skin model, including the epidermis (0.1 mm), dermis (1.5 mm), and subcutaneous fat (8.4 mm). The vertical axis is visually enhanced to clearly depict the thin superficial layers and is not to scale, as indicated by the dimension lines showing true physical thicknesses. The simulation geometry includes a single pencil–beam source and a linear array of 16 detectors on the surface, spanning source-detector separations (SDS) from 0.5 mm to 8.0 mm. Illustrative photon paths (dashed lines) show that light detected at longer SDS has, on average, interrogated deeper tissue volumes.

The simulated optical probe consisted of a single pencil-beam source, positioned at the center of the phantom’s surface and directed perpendicularly into the tissue. An array of 16 circular detectors (0.4 mm diameter) was arranged linearly on the surface to record the reflected light at source-detector separations (SDS) ranging from 0.5 mm to 8.0 mm in 0.5 mm increments. This small diameter was chosen to achieve ideal spatial resolution of the diffuse reflectance profile for foundational optimization, acknowledging that a final device would use larger elements (e.g., 1–2 mm photodetectors) to maximize photon collection. Full simulation geometry is depicted in Figure 1.

### 2.3. Spectrally Resolved Tissue Optical Property Model

To ensure high physiological accuracy, the optical properties of each tissue layer—absorption coefficient ($\mu_{a}$), scattering coefficient ($\mu_{s}$), anisotropy factor (*g*), and refractive index (*n*)—were calculated dynamically as a function of wavelength (*λ*). The baseline properties for the non-vascular components were derived from established literature models [22], treating each tissue layer as a homogeneous effective medium.

The absorption of the epidermis was modeled based on the melanin volume fraction ($f_{mel}$), set to 5% for a representative light skin type. This single-point analysis is justified by the inherent optical properties of the 800 nm wavelength, where melanin absorption is minimized compared to visible wavelengths, providing theoretical robustness to skin tone (as detailed in the Discussion). The corresponding wavelength-dependent absorption coefficient was calculated using the standard formula [22]:

(2) $$\mu_{a,epidermis}(\lambda )=f_{mel}(6.6\times 10^{11}\cdot \lambda^{-3}.^{33})$$

While melanin absorption is complex, this approximation is widely accepted for effective medium models in the visible and NIR range [22,23]. The absorption of the bloodless dermis was modeled as a mixture of a baseline absorber and a 65% water volume fraction (f_water). The reduced scattering coefficient ($\mu_{s}^{\prime }$) for all tissue layers was modeled using a general power-law relationship, which accounts for the wavelength-dependency of scattering from cellular and fibrous structures [22]:

(3) $${\mu^{\prime }}_{s}(\lambda )=A\cdot (\lambda /500{)}^{-B}$$

The specific amplitude (A) and scatter power (B) parameters for each layer were adapted from the literature [23,24]. The full scattering coefficient ($\mu_{s}$) was then calculated from $\mu_{s}^{\prime }$ using the relation $\mu_{s}={\mu^{\prime }}_{s}/(1-g)$. The final calculated baseline properties are provided in Table 2.

The dermis was subsequently modeled as a composite medium containing this bloodless background matrix and a vascular component. The blood volume fraction (BVF) of the dermis is a key parameter influencing sensor sensitivity. Based on established multi-layered skin models [21,25], the typical blood volume in the reticular dermis ranges from 2% to 10%. Therefore, we selected 5% BVF to represent a standard, well-perfused baseline for optimization, and 1% BVF to simulate the severe peripheral vasoconstriction observed in clinical shock states. A preliminary simulation sweep was conducted across a range of physiological BVF values (1–5%) to investigate this dependence. For the final comparative analysis, a robust BVF of 5% was chosen. The effective optical properties of the dermis were calculated using the volume-weighted linear mixture model [22]:

(4) $$\mu_{a,dermis}=\left(1-BVF\right)\cdot \mu_{a,base}+BVF\cdot \mu_{a,blood}$$

(5) $$\mu_{s,dermis}=\left(1-BVF\right)\cdot \mu_{s,base}+BVF\cdot \mu_{s,blood}$$

### 2.4. Advanced Optical Property Model for Whole Blood

A validated, multi-component model was implemented to calculate the optical properties of whole blood as a function of wavelength (λ), hematocrit (Hct), and blood oxygen saturation (SO_2_). This model, detailed below, formed the core of our simulation of hemoconcentration.

#### 2.4.1. Blood Absorption Coefficient (μ_a_)

The absorption coefficient of blood was calculated from first principles based on the definition of the absorption coefficient in a solution:

(6) $$\mu_{a,\lambda }\left(\lambda ,Hct,SO^{2}\right)=2.303\cdot \varepsilon_{eff}\left(\lambda ,SO^{2}\right)\cdot C_{Hb,molar}\cdot Hct$$

The effective molar extinction coefficient ($\varepsilon_{eff}$) in cm^−1^/(mol/L) is a weighted sum of the coefficients for pure oxyhemoglobin ($\varepsilon_{HbO_{2}}$) and deoxyhemoglobin ($\varepsilon_{Hb}$):

(7) $$\varepsilon_{eff}=SO_{2}\cdot \varepsilon_{HbO_{2}}(\lambda )+(1-SO_{2})\cdot \varepsilon_{Hb}(\lambda )$$

Here, $C_{Hb,molar}$ is the molar concentration of hemoglobin within erythrocytes (calculated from a mean corpuscular hemoglobin concentration of 340 g/L and a hemoglobin molar mass of 64,500 g/mol), and Hct is the hematocrit. The constant 2.303 converts from base-10 logarithm (used in extinction coefficients) to natural logarithm (used in absorption coefficients). The spectral data for $\varepsilon_{HbO_{2}}(\lambda )$ and $\varepsilon_{Hb}(\lambda )$ were sourced from the tabulated data compiled by [26].

#### 2.4.2. Blood Scattering Coefficient (μ_s_)

The scattering of whole blood was modeled using a dependent scattering theory validated by Bosschaart et al. [27], which accounts for inter-particle interference effects at high cell densities:

(8) $$\mu_{s,\lambda }\left(\lambda ,Hct\right)=\mu_{s}^{\prime }\left(\lambda \right)\cdot Hct\cdot (1-Hct{)}^{2}$$

**Figure 2:**
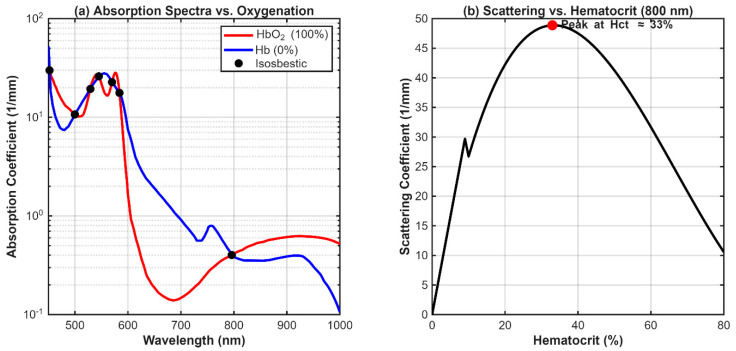
Validation of the Wavelength- and Hematocrit-Dependent Blood Optical Property Model. (**a**) Simulated absorption spectra for whole blood (Hct = 42%) at 100% (HbO_2_) and 0% (Hb) oxygen saturation. The model correctly reproduces the known isosbestic points where the curves intersect, most notably near 584 nm and 796 nm, confirming its spectral accuracy. (**b**) Simulated scattering coefficient (μs) at 800 nm as a function of hematocrit. The curve demonstrates the characteristic non-linear behavior of dependent scattering, with a peak at Hct ≈ 33%, validating the correct implementation of the scattering physics.

Here, $\mu_{s,\lambda }$ is a wavelength-dependent term representing the effective scattering cross-section of a single red blood cell. This term was empirically modeled and calibrated to fit the compiled literature data presented by Bosschaart et al. [27]. This model correctly captures the non-linear relationship between scattering and hematocrit. The implementation of this blood optics model was validated by confirming its ability to reproduce known isosbestic points and the characteristic non-linear scattering dependence on Hct, as shown in Figure 2.

#### 2.4.3. Simulation of Hemoconcentration

To evaluate the proposed sensor’s sensitivity, a clinically significant hemoconcentration event was simulated by defining two distinct clinical states. The first, a “Healthy State,” was defined with a baseline hematocrit of 42%. The second, a “Dengue State,” was defined with a hematocrit of 52.5%, representing a +25% (0.25 × 42% ≈ 10.5% absolute change) relative increase from the healthy baseline. It is explicitly noted that this simulation isolates the optical contribution of the hematocrit rise. It does not model the concurrent increase in interstitial water (edema) associated with plasma leakage, which would further affect the signal at 940 nm. This magnitude of change is consistent with the WHO diagnostic criteria for severe Dengue [1].

A full Monte Carlo simulation was executed for both the “Healthy” and “Dengue” states at each of the four selected wavelengths: 577 nm (visible Hb peak), 660 nm (Hb/HbO_2_ contrast peak), 800 nm (Hb/HbO_2_ isosbestic point), and 940 nm (water absorption peak). Based on the initial performance limitations of the visible wavelengths, the final, high-fidelity comparative simulation (4 × 10^8^ photons) was executed only for the most promising candidates: 800 nm and 940 nm. For all simulations, blood oxygen saturation was fixed at a typical venous level of 75% to maintain a consistent physiological baseline. This value was chosen deliberately to simulate a worst-case scenario for absorption, maximizing the potential confounding effect of SpO_2_ variation and thereby rigorously challenging the robustness of the 800 nm isosbestic point.

The primary metric, sensor sensitivity, was quantified as the absolute percentage change in the total number of detected photons (R) between the two states for each detector at each source-detector separation (SDS):

(9) $$Sensitivity(\%)=|\frac{\left(R_{Dengue}-R_{Healthy}\right)}{R_{Healthy}}|\times 100$$

To quantify statistical reliability, a line representing the 0.81% noise floor is included in sensitivity plots, serving as a practical validity threshold for the detected physiological signal. In addition to reflectance, the mean optical pathlength of detected photons was recorded for each SDS and wavelength to verify the tissue probing depth.

## 3. Results

The simulations were conducted in three stages. First, the influence of the baseline dermal blood volume fraction (BVF) on sensor sensitivity was investigated to establish a robust model parameter. Second, the optical pathlength and penetration depth were analyzed to verify tissue interrogation. Finally, a comparative analysis was performed at the selected BVF to determine the optimal wavelength and source-detector separation (SDS).

### 3.1. Dependence of Sensitivity on Dermal Blood Volume Fraction

**Figure 3:**
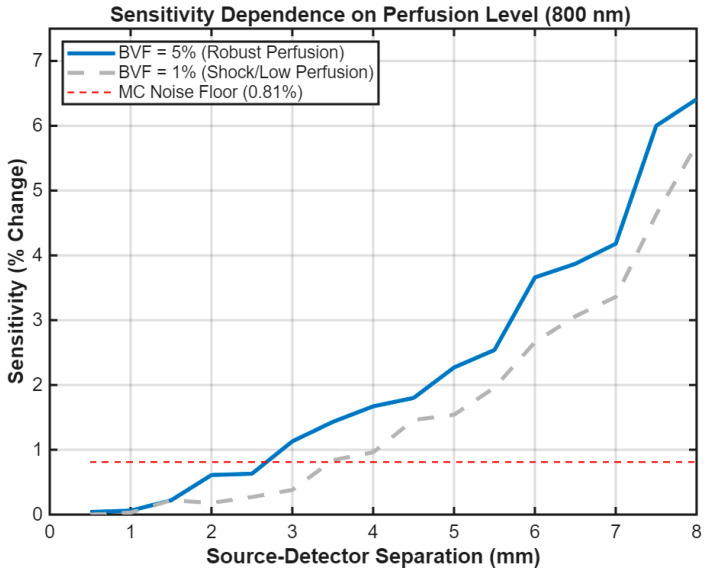
Sensor sensitivity to a +25% relative Hct rise at 800 nm for robust (5% BVF, solid blue) and shock (1% BVF, dashed gray) perfusion. Max sensitivities of 6.41% and 5.71% occur at 8.0 mm, respectively. The red dashed line (0.81%) represents the Monte Carlo stochastic limit; practical hardware with temporal averaging can achieve significantly lower noise floors (<0.1%). Sensitivity exceeding this limit by a factor of >7 confirms sensor viability even during severe vasoconstriction associated with Dengue Shock Syndrome (DSS).

The sensitivity of the optical measurement to hemoconcentration was found to depend on the baseline blood perfusion of the dermis. A preliminary investigation at 800 nm simulated the sensor’s response to a +25% Hct rise at two representative perfusion levels: a moderate perfusion of 1% BVF and a high perfusion of 5% BVF (Figure 3). The results indicate a direct relationship between blood volume and signal strength. The sensor’s response to Hct change was simulated across two representative perfusion extremes: a robust perfusion of 5% BVF 5% BVF and a low perfusion of 1% BVF 1% BVF (Figure 3). The results indicate a direct relationship between blood volume and signal strength. The peak sensitivity for the robust 5% BVF case is 6.41%, and for the low 1% BVF case, it remains high at 5.71%. This indicates that the sensor’s ability to detect hemoconcentration under low-perfusion conditions. Based on these findings, a robust BVF of 5% was selected for the final comparative analysis to ensure the sensor design is optimized for a well-perfused measurement site.

### 3.2. Optical Pathlength and Penetration Depth

**Figure 4:**
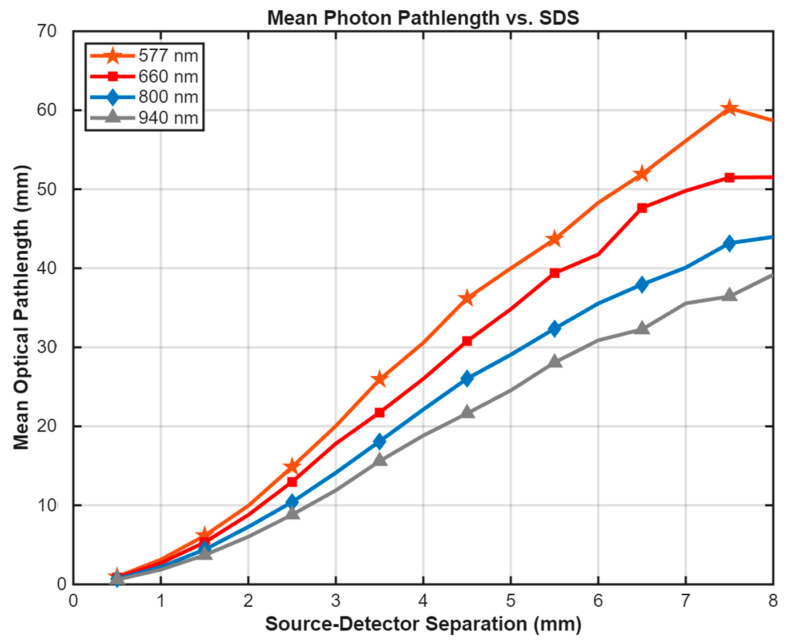
Mean Optical Pathlength of Detected Photons vs. Source-Detector Separation. The plot shows the average pathlength traveled by photons within the tissue for the four tested wavelengths in the healthy baseline state. At the critical separation of 8.0 mm, the 800 nm wavelength (blue) exhibits a mean pathlength of approximately 45 mm, confirming that the detected light undergoes multiple scattering events and deeply interrogates the dermal volume. Note that the apparent long pathlength for 577 nm at large separations is associated with extremely low signal intensity (see Figure 5a).

**Figure 5:**
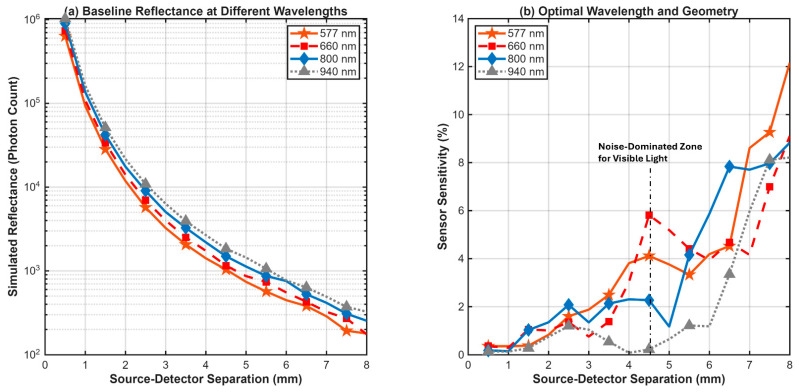
Comparison of Sensor Sensitivity to Hemoconcentration Across Different Wavelengths and Geometries. (**a**) Simulated baseline diffuse reflectance (photon count) for a healthy state (Hct = 42%, BVF = 5%) at the four tested wavelengths. The NIR wavelengths (800 nm, 940 nm) show higher overall reflectance at long SDS, indicating deeper light penetration compared to the visible wavelengths (577 nm, 660 nm). (**b**) The main comparative result, showing the sensor sensitivity (%) as a function of SDS. The 800 nm wavelength demonstrates a robust and significant sensitivity peak at long SDS, outperforming other wavelengths in terms of reliability and practical applicability. Note: Erratic fluctuations in the 577 nm curve for SDS > 4 mm indicate noise dominance and represent the limit of reliable signal detection.

To verify the probing depth of the sensor, the mean optical pathlength of detected photons was calculated for the healthy baseline state (Figure 4). The results confirm that longer source-detector separations correspond to significantly longer interaction paths within the tissue. At the critical SDS of 8.0 mm, the mean pathlength for 800 nm light was approximately 45 mm. This indicates that the detected photons undergo hundreds of scattering events, ensuring broad interrogation of the dermal volume. While the 577 nm wavelength shows a superficially long pathlength at large separations, this is an artifact of high absorption where only photons traveling tortuous, shallow paths survive, as evidenced by the extremely low signal intensity in Figure 5a.

### 3.3. Wavelength and Geometry Optimization

The primary results of the comparative study are summarized in the two-panel Figure 5. Panel 5(a) displays the simulated baseline diffuse reflectance for the healthy state (Hct = 42%, BVF = 5%) at the four tested wavelengths. A clear distinction is observed between the visible and near-infrared (NIR) regimes. The NIR wavelengths (800 nm and 940 nm) exhibit significantly higher overall reflectance, particularly at longer SDS, indicating deeper light penetration. Conversely, the 577 nm signal is the most strongly attenuated, dropping by several orders of magnitude more than the NIR signals due to the combined high absorption of melanin in the epidermis and hemoglobin in the dermis.

Panel 5(b) presents the main finding of the study: the comparative sensor sensitivity for each wavelength as a function of SDS. The results show that sensitivity is minimal at short SDS (<2 mm) for all wavelengths, confirming that shallow-probing geometries are ineffective for monitoring the dermis. All wavelengths show a general trend of increasing sensitivity at longer SDS. The visible wavelengths (577 nm and 660 nm) demonstrate peak sensitivities of 11.1% and 8.2% respectively, but the signal at 577 nm is potentially unstable due to low photon counts. Specifically, the erratic peaks for 577 nm at SDS > 4 mm (Figure 5b) reflect stochastic noise dominance, identifying the practical reliability threshold for this wavelength. The 940 nm wavelength showed a peak sensitivity of 8.4%. The 800 nm wavelength shows a stable and consistent trend, with sensitivity reaching 6.4% at a separation of 8.0 mm. A consolidated performance comparison across all evaluated wavelengths and geometries is presented in Table 3 While this magnitude is lower than the theoretical maximum of visible light, it represents a robust, measurable signal that is less susceptible to surface artifacts. To confirm this robustness, a theoretical calculation of absorption change (Δμ_a_) was performed for Hct rise versus a maximal SpO_2_ swing (75% → 98%). The resulting SpO_2_ absorption change at 660 nm was calculated to be 2.56 times the target Hct signal, confirming strong confounding. Conversely, SpO_2_ absorption at 800 nm was found to be only 6% of the target Hct signal, definitively supporting the selection of the 800 nm isosbestic point.

## 4. Discussion

The goal of this study was to identify an optimal wavelength and source-detector geometry for the non-invasive monitoring of Dengue-related hemoconcentration. Our high-fidelity computational model, which incorporated validated and spectrally resolved optical properties, provides a clear, data-driven recommendation for the design of a practical clinical device.

A significant finding of this work is that the sensitivity of a DRS measurement is non-linearly dependent on the tissue’s baseline perfusion level. As shown in Figure 3, the sensitivity of the sensor scales with the local blood volume fraction. As shown in Figure 3, a highly perfused measurement site (5% BVF) yields a stronger signal change (6.4%) compared to a moderately perfused site (1% BVF, 5%). This suggests that sensor placement on well-vascularized skin regions will enhance performance. This finding is clinically relevant, indicating that sensor performance may vary with measurement site (e.g., forearm vs. fingertip). However, the robust sensitivity at a high BVF of 5% confirms the technique’s viability across a range of physiological conditions.

The main comparative analysis (Figure 5) clearly demonstrates the trade-offs between different optical regimes. While visible wavelengths like 577 nm and 660 nm offer high theoretical sensitivity (peak > 11%), their practical utility is severely limited. The high attenuation from epidermal melanin and hemoglobin (Figure 5a) leads to an extremely low photon count at the detector, causing the 577 nm and 660 nm signals to fall below the simulation noise floor at long SDS. This validity threshold failure makes them practically unfeasible for real hardware applications, despite their high theoretical sensitivity.

The robustness of the 800 nm selection against melanin is supported by two physical principles. First, at 800 nm, the melanin absorption coefficient ($\mu_{a,\mathrm{mel}}$) is inherently low compared to visible light, significantly reducing its influence on deeper tissue interrogation. Second, the sensor’s sensitivity metric is defined as a relative percentage change in reflectance (ΔR/R). This ratiometric approach makes the sensitivity largely invariant to the static surface attenuation caused by melanin, provided the detected signal is above the noise floor. This combination of NIR wavelength selection and ratiometric measurement ensures that the sensor’s performance is robust across various skin phototypes, a critical requirement for global deployment. However, we acknowledge a practical limitation regarding signal magnitude. While 800 nm is robust to melanin absorption (preserving sensitivity), extreme pigmentation (Fitzpatrick VI) will significantly attenuate the absolute reflectance intensity. In such cases, the detected signal may approach the noise floor, requiring higher optical source power to maintain an adequate signal-to-noise ratio [15].

In contrast, the near-infrared wavelength of 800 nm emerges as the most suitable candidate among the four wavelengths tested. Its position in the “optical window” allows for deep penetration past the epidermis, enabling effective interrogation of the dermal vascular plexus. This is confirmed by the optical pathlength analysis in Figure 5, which shows that 800 nm light detected at a 7.0 mm separation has traveled an average of ~40 mm through the tissue, ensuring extensive interaction with the dermal volume. While a longer mean pathlength suggests increased interaction with the tissue volume, it does not guarantee depth. The combination of increased pathlength at larger separations and the observed low sensitivity at short separations is consistent with, but does not prove, greater dermal contribution. This substantial pathlength relative to the source-detector separation (DPF≈5.7) acts as an optical amplifier, ensuring that even the relatively low absorption of hemoglobin at 800 nm produces a substantial and measurable signal change.

Most importantly, 800 nm provides a substantial and reliable sensitivity of 5.2% to a clinically significant hemoconcentration event. The sensitivity peak occurs at a long SDS (7.0 mm), confirming that deep-probing geometries are essential for this application.

A key consideration for any optical sensor is whether the predicted physiological signal exceeds the noise floor of the instrument. Our simulations predict a 5.2% change in reflectance at 800 nm for a clinically significant hematocrit rise. When accounting for dominant noise sources, including shot noise, detector dark noise, and analog-to-digital quantization, the simulated system noise level (~1.6%) remains well below the predicted reflectance change (>5%). Moreover, with an LED source operating near 800 nm and milliwatt-level optical power, the resulting photon budget at a source–detector separation of ~7 mm is expected to support sub-percent repeatability with appropriate signal averaging, indicating strong theoretical feasibility for experimental realization. However, it is important to note that these sensitivity curves represent single-point estimates based on fixed model assumptions. The 0.81% noise floor reflects stochastic simulation noise and does not account for physiological uncertainty or the anatomical variability present in a real-world population. Therefore, these results should be interpreted as demonstrating general design trends rather than absolute quantitative precision.

The key advantage of 800 nm is its robustness against physiological confounders. To quantify this, a theoretical analysis was conducted to compare the change in blood’s absorption coefficient ($\Delta \mu_{a}$) from hemoconcentration versus that from a physiological swing in blood oxygen saturation (SpO_2_ from 75% to 98%). At 660 nm, the SpO_2_-induced $\Delta \mu_{a}$ was 2.56 times larger than the target Hct signal, confirming it as a major confounder. Conversely, at the 800 nm isosbestic point, the SpO_2_-induced $\Delta \mu_{a}$ was only 6% of the Hct signal. This quantitatively demonstrates that 800 nm effectively isolates the measurement of hemoconcentration from the influence of blood oxygenation. The complete quantitative results of our comparative analysis are presented in Section 3 and summarized in Table 4.

This study relies on a simplified multi-layered tissue model. While the chosen 1.5 mm dermal thickness is representative of sites like the forearm, anatomical variations exist. A supplementary simulation with a reduced dermal thickness of 1.0 mm (representative of fingertips) yielded a sensitivity of 6.7%, confirming that the sensor’s performance is robust to these anatomical variations. While this effective medium approach is standard for determining baseline design parameters, it does not capture the heterogeneity of the discrete vascular network or the dynamic effects of pulsatile blood flow. The simulated depth sensitivity is therefore interpreted as an average interrogation of the vascular bed rather than a pinpoint measurement. However, since hemoconcentration is a slow, systemic physiological change, the sensor is designed to monitor the static (DC) baseline reflectance rather than the pulsatile (AC) photoplethysmogram. We explicitly acknowledge that DC measurements in wearable devices are prone to instability from motion, thermal drift, and fiber-optic coupling variations. Therefore, the presented DC sensitivity must be complemented by hardware solutions that continuously track and compensate for these sources of noise. To mitigate coupling artifacts and baseline drift in DC measurements, a short-separation reference channel (e.g., 2 mm) can be implemented to regress superficial noise.

Additionally, the simulation assumes a monochromatic light source. In practice, light-emitting diodes (LEDs) have a spectral bandwidth and Lambertian angular divergence, which means the current model may overestimate the effective probing depth. Lambertian angular divergence, which means the current model may overestimate the effective probing depth. This will be addressed in future hardware validation. Additionally, we acknowledge that a physical photodetector (e.g., 2 mm diameter) would integrate light over a larger area, improving SNR via averaging but potentially broadening the spatial sensitivity profile compared to the virtual 0.4 mm detector modeled here.

Finally, while we modeled a fixed water content, we acknowledge that plasma leakage is the cause of hemoconcentration, and this leakage leads to edema, potentially altering tissue scattering and water absorption. The confounding effect of increased interstitial water on the 940 nm wavelength is a known limitation of our fixed-parameter in silico study. Future designs could leverage a dual-wavelength approach (800 nm and 940 nm) to simultaneously estimate hematocrit and tissue water content, allowing for edema compensation. Future work will also focus on validating these findings in tissue-mimicking phantoms and, subsequently, on human subjects. The current model assumes perfect optical coupling between the probe and the skin. In a practical wearable device, contact pressure variations, motion artifacts, and air gaps introduce significant measurement noise. To mitigate this practical limitation, the final sensor must incorporate a robust mechanical design for stable coupling or a dual-channel architecture to regress this superficial noise [15]. Despite these limitations, the presented in silico model provides a quantitative foundation for sensor design and serves as a basis for future experimental validation and device development.

## 5. Conclusions

This computational investigation successfully determined optimized design parameters for a non-invasive optical sensor for hemoconcentration. The simulations indicate that, within the evaluated spectral set, an operating wavelength of 800 nm combined with a source-detector separation of ≥6.0 mm or greater provides the optimal balance of sensitivity, tissue penetration depth, and robustness against physiological confounders like melanin and blood oxygenation. Theoretical analysis confirms that 800 nm minimizes epidermal melanin absorption compared to visible wavelengths, supporting its suitability for diverse skin phototypes. We acknowledge that the optimal SDS imposes a form-factor constraint for compact and pediatric devices, representing a performance/size trade-off. However, this separation is necessary to achieve the robust 6.00% signal required for high-fidelity DC sensing. These findings provide a clear, evidence-based directive for the development and prototyping of a clinical device for the management of severe Dengue.

The introduced work could enable the design of a sensor that continuously monitors hemoconcentration in Dengue patients, facilitating the early detection of plasma leakage. The immediate next step is the experimental validation of these simulation results using liquid tissue phantoms with independently tunable optical properties (Hct, BVF, and scattering) to confirm the optimal geometry. Subsequently, miniaturization into a wearable device for continuous bedside monitoring in resource-limited, dengue-endemic regions will occur.

## Figures and Tables

**Table 1:** Signal-to-Noise Ratio (SNR) Convergence Study. Simulations were performed at 800 nm for a source-detector separation of 6.0 mm. SNR was calculated based on 10 repeated runs for each photon count.

Photon Count	SNR (dB)	Noise Level (%)
$1\times 10^{6}$	10.32	30.47%
$1\times 10^{7}$	18.83	11.45%
$5\times 10^{7}$	27.77	4.09%
$1\times 10^{8}$	35.84	1.62%
$2\times 10^{8}$	35.50	1.68%

**Table 2:** Optical Properties Used for the In Silico Skin Tissue Model. The values for the bloodless tissue components were generated using established literature models [22,23,24]. The parameter n denotes the refractive index.

Wavelength (nm)	Tissue Layer	µa (1/mm)	µs (1/mm)	g	n
577	Epidermis (5% mel)	21.08	201.66	0.8	1.37
	Dermis (Base)	0.02	168.42	0.85	1.37
	Subcutaneous Fat	0.01	53.50	0.75	1.45
660	Epidermis (5% mel)	13.47	164.84	0.8	1.37
	Dermis (Base)	0.02	143.33	0.85	1.37
	Subcutaneous Fat	0.01	48.05	0.75	1.45
800	Epidermis (5% mel)	7.09	123.53	0.8	1.37
	Dermis (Base)	0.02	113.79	0.85	1.37
	Subcutaneous Fat	0.01	41.19	0.75	1.45
940	Epidermis (5% mel)	4.15	96.98	0.8	1.37
	Dermis (Base)	0.02	93.77	0.85	1.37
	Subcutaneous Fat	0.01	36.21	0.75	1.45

**Table 3:** Summary of Simulated Sensor Performance for Hemoconcentration Detection.

Wavelength (nm)	Peak Sensitivity (%)	Optimal SDS (mm)	Key Advantages/Disadvantages
577	11.05	7	Adv: High hemoglobin absorption contrast. Disadv: Unstable signal, severe melanin interference, shallow penetration.
660	8.17	8	Adv: High sensitivity. Disadv: Strong confounder from blood oxygenation (SpO_2_), moderate melanin interference.
800	6.41	8	Adv: Robust and reliable sensitivity, deep penetration, insensitive to SpO_2_ (isosbestic point), low melanin interference.
940	8.42	8	Adv: Deepest penetration. Disadv: Sensitivity compromised by background water absorption.

**Table 4:** Quantitative comparison of blood absorption coefficient changes ($\Delta \mu_{a}$) at 660 nm and 800 nm due to hemoconcentration (+25% relative Hct rise) versus a physiological $SpO_{2}$ swing (75% to 98%).

Wavelength	$\Delta \mu_{a}$ from Hct Rise $\left(mm^{-1}\right)$	$\Delta \mu_{a}\;\mathrm{from}\;SpO_{2}$ Swing $\left(mm^{-1}\right)$	Interference Ratio $(SpO_{2}$/Hct)
660 nm	0.052	0.133	2.56
800 nm	0.048	0.003	0.06 (6%)

## Data Availability

The data generated in this study consist of Monte Carlo simulation outputs and derived sensitivity metrics. These data are available from the corresponding author upon reasonable request.

## References

[1] Simmons C.P., Farrar J.J., van Vinh Chau N., Wills B. (2012). Dengue. N. Engl. J. Med..

[2] El Kabbani S., Saleh G. (2026). Next—Generation Diagnostic Technologies for Dengue Virus Detection: Microfluidics, Biosensing, CRISPR, and AI Approaches. Sensors.

[3] Sivasubramanian K., Bharath R.R., Vajravelu L.K., Kumar D.M., Banerjee A. (2025). Key Laboratory Markers for Early Detection of Severe Dengue. Viruses.

[4] Thapa B., Lamichhane P., Shrestha T., Lamichhane S., Karki S., Pradhananga S., Batajoo K.H., Pudasaini P. (2025). Leukopenia and thrombocytopenia in dengue patients presenting in the emergency department of a tertiary center in Nepal: A cross-sectional study. BMC Infect. Dis..

[5] World Health Organization (2009). Dengue Guidelines for Diagnosis, Treatment, Prevention and Control.

[6] Rahat F., Khanam M., Iman K., Ghosh U., Ghosh N. (2021). Importance of Platelet Count and Hematocrit in Dengue Fever in Children. Bangladesh J. Child Health.

[7] Alsunaidi B., Althobaiti M., Tamal M., Albaker W., Al-Naib I. (2021). A review of non-invasive optical systems for continuous blood glucose monitoring. Sensors.

[8] Althobaiti M. (2023). Estimation of the Differential Pathlength Factor for Human Skin Using Monte Carlo Simulations. Diagnostics.

[9] Berkow L., Rotolo S., Mirski E. (2011). Continuous noninvasive hemoglobin monitoring during complex spine surgery. Anesth. Analg..

[10] Taylor-Williams M., Spicer G., Bale G., Bohndiek S.E. (2022). Noninvasive hemoglobin sensing and imaging: Optical tools for disease diagnosis. J. Biomed. Opt..

[11] Lima A., Bakker J. (2005). Noninvasive monitoring of peripheral perfusion. Intensive Care Med..

[12] Talke P., Stapelfeldt C. (2006). Effect of peripheral vasoconstriction on pulse oximetry. J. Clin. Monit. Comput..

[13] Moulton S.L., Mulligan J., Srikiatkhachorn A., Kalayanarooj S., Grudic G.Z., Green S., Gibbons R.V., Muniz G.W., Hinojosa-Laborde C., Rothman A.L. (2016). State-of-the-art monitoring in treatment of dengue shock syndrome: A case series. J. Med. Case Rep..

[14] Farrell T.J., Patterson S.M. (1992). A diffusion theory model of spatially resolved, steady-state diffuse reflectance for the noninvasive determination of tissue optical properties in vivo. Med. Phys..

[15] Althobaiti M., Al-Naib I. (2021). Optimization of Dual-Channel Near-Infrared Non-Invasive Glucose Level Measurement Sensors Based On Monte-Carlo Simulations. IEEE Photonics J..

[16] Althobaiti M. (2022). In Silico Investigation of SNR and Dermis Sensitivity for Optimum Dual-Channel Near-Infrared Glucose Sensor Designs for Different Skin Colors. Biosensors.

[17] Scholkmann F., Kleiser S., Metz A.J., Zimmermann R., Pavia J.M., Wolf U., Wolf M. (2014). A review on continuous wave functional near-infrared spectroscopy and imaging instrumentation and methodology. Neuroimage.

[18] Jenie R.P., Nasiba U., Rahayu I., Nurdin N.M., Husein I., Alatas H. (2019). Review on wavelength for non-invasive blood hemoglobin level measurement optical device. AIP Conf. Proc..

[19] Fang Q., Boas D.A. (2009). Monte Carlo simulation of photon migration in 3D turbid media accelerated by graphics processing units. Opt. Express.

[20] Yuan Y., Yu L., Doğan Z., Fang Q. (2018). Graphics processing units-accelerated adaptive nonlocal means filter for denoising three-dimensional Monte Carlo photon transport simulations. J. Biomed. Opt..

[21] Bashkatov A.N., Genina E.A., Kochubey V.I., Tuchin V.V. (2005). Optical properties of human skin, subcutaneous and mucous tissues in the wavelength range from 400 to 2000 nm. J. Phys. D Appl. Phys..

[22] Jacques S.L. (2013). Optical properties of biological tissues: A review. Phys. Med. Biol..

[23] Jonasson H., Fredriksson I., Bergstrand S., Östgren C.J., Larsson M., Strömberg T. (2023). Absorption and reduced scattering coefficients in epidermis and dermis from a Swedish cohort study. J. Biomed. Opt..

[24] Verdel N., Marin A., Milanič M., Majaron B. (2019). Physiological and structural characterization of human skin in vivo using combined photothermal radiometry and diffuse reflectance spectroscopy. Biomed. Opt. Express.

[25] Meglinski I.V., Matcher S.J. (2002). Quantitative assessment of skin layers absorption and skin reflectance spectra simulation in the visible and near-infrared spectral regions. Physiol. Meas..

[26] Prahl S. Optical Absorption of Hemoglobin. Oregon Medical Laser Center. (n.d.).. http://omlc.org/spectra/hemoglobin/index.html.

[27] Bosschaart N., Edelman G.J., Aalders M.C.G., Van Leeuwen T.G., Faber D.J. (2014). A literature review and novel theoretical approach on the optical properties of whole blood. Lasers Med. Sci..

